# *Corynebacterium* ocular infection after Baerveldt glaucoma implant surgery: treatment involving immediate tube withdrawal and temporary subconjunctival tube placement: a case report

**DOI:** 10.1186/s12886-021-02136-6

**Published:** 2021-10-18

**Authors:** Naruka Mitsui, Kae Sugihara, Jiro Seguchi, Etsuo Chihara, Yuki Morizane, Akiko Narita

**Affiliations:** 1 Department of Ophthalmology, Okayama Saiseikai General Hospital, 2-25 Kokutaicho, Kita-ku, Okayama, 700-8511 Japan; 2 Sensho-kai Eye Institute, 50-1 Minamiyama, Kyoto, Iseda, Uji, Kyoto, 611-0043 Japan; 3 Department of Ophthalmology, Okayama University Graduate School of Medicine, Dentistry and Pharmaceutical Science, 2-5-1 Shikata-cho, Kita-ku, Okayama, 700-8558 Japan

**Keywords:** Glaucoma drainage device, Tube exposure, Endophthalmitis, Corynebacterium, Case report, Ocular infection

## Abstract

**Background:**

We report a case of *Corynebacterium* endophthalmitis secondary to tube exposure following Baerveldt glaucoma implant surgery that was successfully treated with prompt tube withdrawal and temporary subconjunctival tube placement without removing the glaucoma drainage device.

**Case presentation:**

A 65-year-old Japanese man with secondary glaucoma underwent glaucoma drainage device surgery with a donor scleral patch graft in the inferonasal quadrant of his right eye. Ten months after surgery, he presented with tube exposure due to dehiscence of the overlying conjunctiva and erosion of the scleral patch graft. Eleven days later, mild inflammation was found in the anterior chamber and anterior vitreous body, with the root of the tube surrounded by a plaque at the site of insertion in the anterior chamber. He was diagnosed with infectious endophthalmitis secondary to tube exposure. Two days later, since medical therapy was ineffective, the tube was withdrawn from the anterior chamber and irrigated with a polyvinyl alcohol-iodine solution, and the tube was tucked into the subconjunctival space. Complete resolution of the infection was achieved 1.5 months later. The tube was reinserted nasally into the anterior chamber and covered with a scleral patch graft and a free limbal conjunctival autograft. Thereafter, there has been no recurrence of infection or tube exposure. Twenty eight months after tube reinsertion, his right best-corrected visual acuity was 20/50 and intraocular pressure was 12 mmHg.

**Conclusion:**

Prompt tube withdrawal and temporary subconjunctival tube placement followed by tube reinsertion may be effective for endophthalmitis associated with tube exposure after glaucoma drainage device surgery.

## Background

Tube exposure is a major cause of endophthalmitis after glaucoma drainage device (GDD) surgery, and the reported rate of endophthalmitis following GDD tube exposure is 0.9 to 6.3% [1]. Whether GDDs should be removed or left in place during the treatment of endophthalmitis remains a debatable topic [2]. Infected tubes may serve as a reservoir for the pathogen. However, if the GDD is removed, the large conjunctival scar may hamper subsequent glaucoma surgery.

Here we report a case involving an elderly man with *Corynebacterium* endophthalmitis secondary to tube exposure following Baerveldt glaucoma implant surgery that was successfully treated by prompt tube withdrawal and temporary subconjunctival tube placement without the removal of GDD.

## Case presentation

A 65-year-old Japanese man with a 25-year history of glaucoma secondary to idiopathic uveitis in his right eye and had been treated with several classes of glaucoma medications was referred to our hospital for further consultation. He had cataracts in both eyes, and did not have other ocular diseases, such as uveitis or glaucoma in his left eye. He had a history of renal cancer at the age of 52 years and had systemic hypertension. He reported a 36-year history of smoking (approximately 60 cigarettes per day). He had undergone three mitomycin C-augmented trabeculectomies and two bleb revision procedures, with a history of unspecified bleb-related infection 12 years prior in his right eye. The best-corrected visual acuity (BCVA) for his right eye was 20/100, with intraocular pressure (IOP) of 30 mmHg on five classes of glaucoma medications. His angle was 360° closed by peripheral anterior synechiae, and his kinetic visual field defects were Aulhorn-Greve grade V. Because of the extensive surgical scars in the superior hemisphere due to the multiple glaucoma surgeries and prior bleb-related infection, we decided to implant a Baerveldt glaucoma implant (BG101–350, Johnson & Johnson, Tokyo, Japan) in the inferonasal quadrant, in combination with clear corneal phacoemulsification, aspiration and intraocular lens implantation in February 2018. The silicone tube was ligated near the plate using 7–0 VICRYL® (Ethicon Inc., Somerville, NJ, USA), inserted into the anterior chamber (AC) and covered with a full-thickness donor scleral patch graft. Two venting slits were created using a 7–0 VICRYL® needle proximal to the ligation. His post-surgical IOP was decreased to low-teens with four classes of glaucoma medications.

**Fig. 1:**
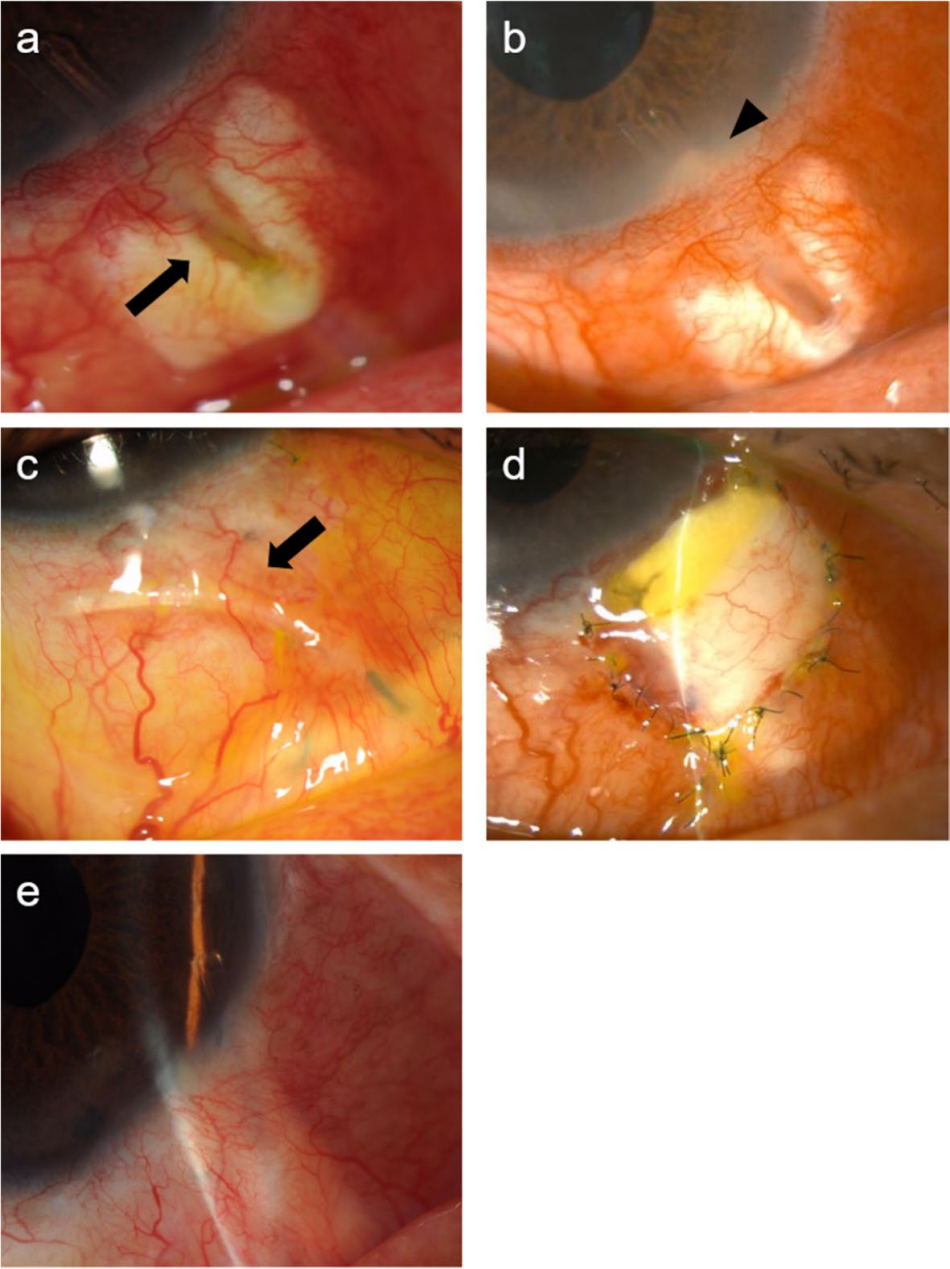
Postoperative findings for a patient with *Corynebacterium* endophthalmitis after Baerveldt glaucoma implant surgery. **a**. Tube exposure (arrow) due to the dehiscence of the overlying conjunctiva and erosion of the scleral patch graft in the inferonasal quadrant of the right eye. **b**. Plaque (arrowhead) surrounding the root of the tube in the anterior chamber. **c**. Tube tucked temporarily into the subconjunctival space (arrow). **d**. Tube reinserted nasally into the anterior chamber and covered with donor sclera and a free conjunctival autograft. **e**. No recurrence of infection or tube erosion at 28 months after tube reinsertion. The patient’s best-corrected visual acuity was 20/50, and intraocular pressure was 12 mmHg in his right eye

Ten months after the surgery, his right AC was clear, BCVA was 20/50, and IOP was 12 mmHg. However, the tube was exposed in the inferonasal quadrant, and dehiscence of the overlying conjunctiva and erosion of the scleral patch graft were evident (Fig. 1a). Since we didn’t find any signs of infection in the AC or surrounding area of the tube, we started topical gatifloxacin 0.3% (Senju Pharmaceutical, Osaka, Japan) immediately after diagnosis and waited for the arrival of ordered donor sclera.

**Fig. 2:**
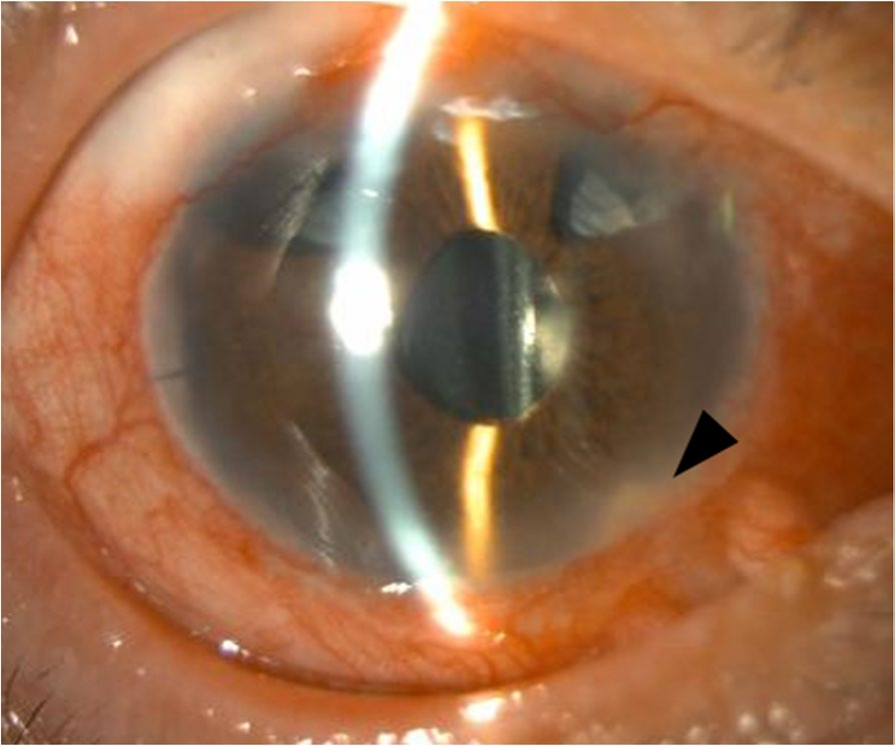
Photograph of the anterior segment of the patient’s right eye when endophthalmitis was diagnosed. Slit-lamp examination revealed conjunctival and scleral hyperemia, grade 3 cells and plaque surrounding the root of the tube (arrowhead) in the anterior chamber, and grade 1 cells in the anterior vitreous body 11 days after tube exposure

Eleven days after tube exposure, at the time of the preoperative examination, we found signs of endophthalmitis in his right eye, in which there were grade 3 cells in the AC and grade 1 cells in the anterior vitreous body (Fig. 2). The root of the tube was surrounded by a plaque at the site of insertion in the AC (Fig. 1b). The patient was diagnosed with infectious endophthalmitis secondary to tube exposure in December 2018. Slit-lamp examination showed that the tube was clear of purulence posterior to the plaque and the bleb was clear and translucent; we determined that the infection had not spread to the bleb yet. Topical cefmenoxime 0.5% (Senju Pharmaceutical, Osaka, Japan) and gatifloxacin 0.3% administered every 2 h were not effective.

Two days after diagnosis of endophthalmitis, the scleral patch graft was removed, and the exposed tube and surrounding area were sterilized using 10 mL of 0.025% polyvinyl alcohol-iodine (PA·IODO Ophthalmic and Eye washing Solution, Nitten Pharmaceutical, Nagoya, Japan) diluted with physiological saline. The silicone tube was withdrawn from the AC following confirmation of the absence of purulent exudation inside the tube. The specimens of the aqueous humor from the AC, which was the main inflammation site in this case, were obtained immediately after tube removal for culture testing. The removed tube and exposed area were vigorously irrigated with 20 mL of 0.025% polyvinyl alcohol-iodine solution. The site of the tube entry was sutured with 8–0 VICRYL® and no leakage from the AC was confirmed. Then, AC was irrigated with vancomycin 20 μg/mL (Shionogi, Osaka, Japan) and ceftazidime 40 μg/mL (GlaxoSmithKline, Tokyo, Japan). After that, the tube was tucked into the subconjunctival space (Fig. 1c). Medical therapy, including topical cefmenoxime 0.5% and gatifloxacin 0.3% administered every 2 h, was continued.

Culture testing identified the growth of *Corynebacterium* species on the specimens of the aqueous humor of his right eye. Susceptibility test results indicated that *Corynebacterium* species were sensitive to penicillin, imipenem, minomycin, gentamicin, and erythromycin, with intermediate sensitivity to levofloxacin and cephem. We added ofloxacine ointment 0.3% (Santen Pharmaceutical, Osaka, Japan) application at bedtime.

**Fig. 3:**
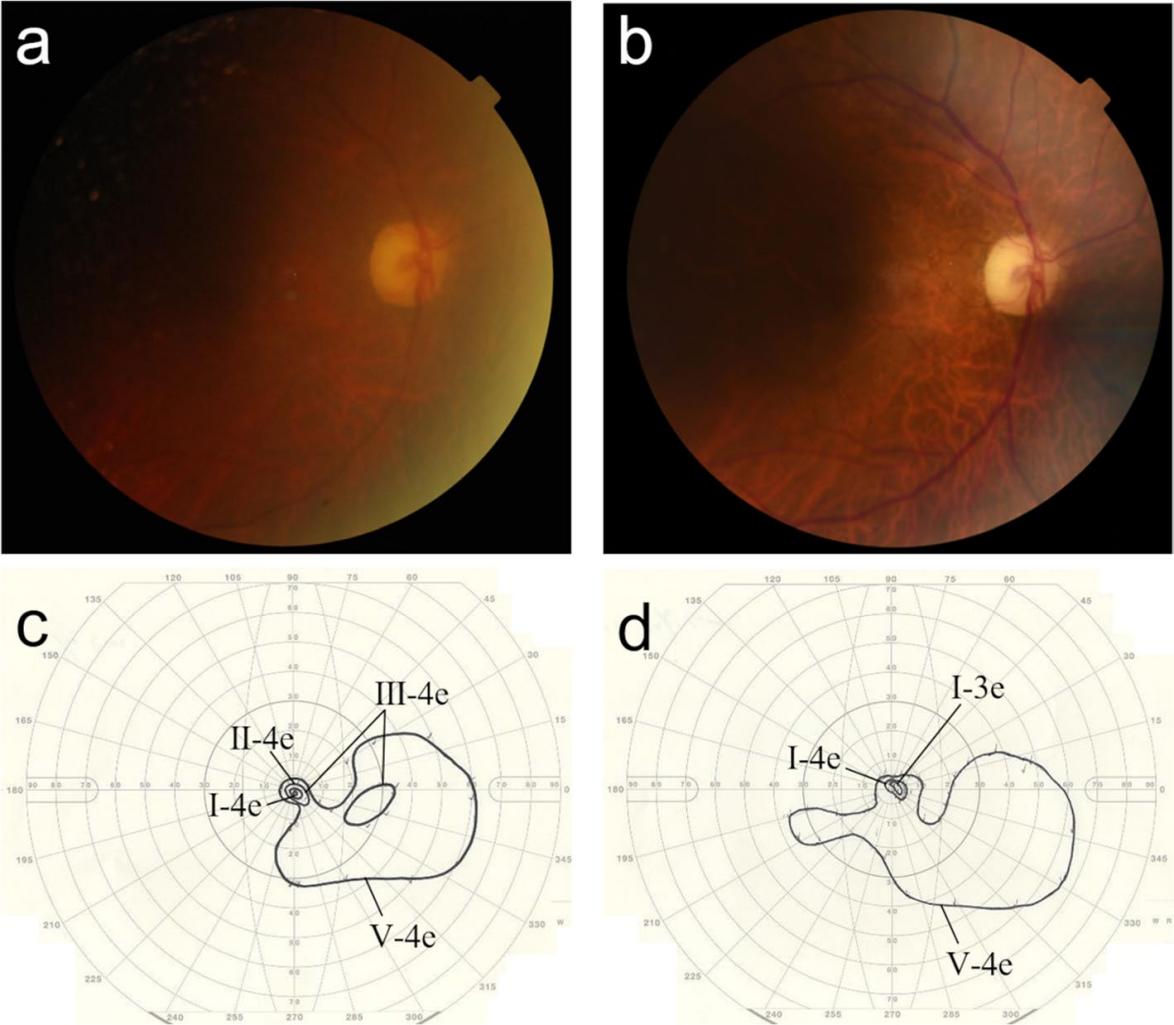
Fundus photographs and kinetic visual field test results of the patient’s right eye. Despite the ocular infection and following IOP fluctuation, the optic nerve head and kinetic visual field of his right eye were maintained at the last visit (**b**, **d**), when compared to those at the first visit (**a**, **c**)

Even though the IOP ranged from 1 to 42 mmHg in his right eye after tube withdrawal, the length of time with the IOP over 30 mmHg was confined to five days with glaucoma medications. The infection completely resolved 1.5 months after tube removal. Thereafter, the tube was reinserted into the AC in the superior-nasal direction so that we could reduce the risk of further tube exposure and infection by avoiding contact between the tube and inferior eyelid. And then the tube was covered with a scleral patch graft and a free limbal conjunctival autograft harvested from his left eye in January 2019 (Fig. 1d). Topical antibiotics were discontinued 1 month after the tube reinsertion. There has been no recurrence of infection or tube exposure since then, although a reduction in the size of the donor sclera was noted (Fig. 1e). His right BCVA was 20/50 and IOP was 12 mmHg on two classes of glaucoma medications in May 2021. Fundus photographs and kinetic visual field test results at the first and last visits demonstrated that the patient’s visual function had been maintained despite the ocular infection and following IOP fluctuation (Fig. 3).

## Discussion and conclusions

Previous reports have shown that *Streptococcus* species and *Haemophilus influenzae* are commonly isolated from endophthalmitis occurring after GDD surgery [2, 3]. *Corynebacterium* species are minor pathogens that are ubiquitous in the environment [4] but may cause endophthalmitis after ocular surgeries [5, 6]. They account for approximately 1% of all causative organisms for endophthalmitis after cataract surgery in the endophthalmitis vitrectomy study [5]. To our knowledge, this is the first report of *Corynebacterium* endophthalmitis occurring after GDD surgery.

Tube exposure, which occurs in 2–5% of cases after GDD implantation [7, 8], is a major cause of endophthalmitis, with reported risk factors including ocular inflammation, steroid use, prior ocular surgery, concomitant surgery, inferior quadrant placement of GDDs, and smoking [9]. In the present case, previous ocular inflammation, multiple prior ocular surgeries, concomitant surgery, inferior quadrant placement of GDD, and smoking history may have predisposed the patient to tube exposure.

Treatment for endophthalmitis secondary to tube exposure remains controversial. Endophthalmitis may be successfully treated by prompt repair of tube exposure with good tissue coverage procedures such as double-thickness pericardial patch grafting, as well as intravitreal injection of antibiotics, even without removal of GDDs [10]. In contrast, Gedde et al. [11] and Perkins et al. [12] recommended GDD removal because infected GDDs may serve as a reservoir for infectious organisms. When the inner lumen of the silicone tube is contaminated through the venting slits of the tube or because of advanced infection in the AC, the risk of infectious organisms spreading to the reservoir is high. In such cases of contamination, the GDDs should be removed to avoid involvement of the entire eye. However, according to our clinical experience, the infection spreads from the exposed area to the AC along the outer surface of the silicone tube. In our case, we found plaque surrounding the root of the silicone tube at the site of insertion in the AC without any leakage from the tube, which was confirmed by slit-lamp microscopy using fluorescein before the surgery. This suggests that the pathogen was a “weak” infectious organism that entered the AC through the outer surface of the silicone tube. Thus, it is likely that the inner lumen of the tube was not infected, and that tube withdrawal, complete tube disinfection with a polyvinyl alcohol-iodine solution, tucking of the tube into the subconjunctival space, and tube reinsertion after the alleviation of inflammation is an effective option.

However, the risk of reinfection cannot be denied [13], and clinicians must pay attention to signs of reinfection. Timing of tube removal may be an important prognostic factor. In a case reported by Fanous and Cohn [13], the tube was removed from the AC and placed in the subconjunctival space without elimination of the Molteno implant after 2 months of medical treatment with antibiotic eye drops and intracameral injection. Endophthalmitis recurred 3 months after reinsertion of the tube, and the Molteno implant was ultimately eliminated. Infectious organisms had likely spread to the subconjunctival space surrounding the GDD before tube withdrawal in their case. Prompt tube withdrawal, complete disinfection, and subsequent tube reinsertion most likely contributed to good IOP control and visual acuity preservation in our case.

The surgical treatment described in this report applies to cases of infectious endophthalmitis secondary to tube exposure following GDD with a tube long enough to reposition, before the infectious organisms spread to the subconjunctival space surrounding the GDD. It is necessary to confirm by slit lamp microscopic inspection that the lumen of the tube, as well as the bleb over the plate, is not infected before surgical treatment. During surgery, small dissection with care to retain as much conjunctiva as possible is recommended. Nevertheless, since the exposed tube is likely to cause epithelial ingrowth of the surrounding conjunctiva, wide resection is needed when there is a prolonged interval between tube exposure and surgical treatment. In cases where there is insufficient conjunctiva to cover the tube, a free limbal conjunctival autograft can be used.

In conclusion, the findings from this case suggest that prompt tube withdrawal from the AC and temporary subconjunctival tube placement followed by tube reinsertion may be an effective treatment for infectious endophthalmitis associated with tube exposure after GDD surgery.

### Patient perspective

The patient was content with the recovery of vision in the right eye, which has enabled him to continue his daily activities.

## Data Availability

Not applicable.

## Abbreviations

GDD: glaucoma drainage device
BCVA: best-corrected visual acuity
IOP: intraocular pressure
AC: anterior chamber
